# Narrative dataset of fiscal actions in the EU new member states

**DOI:** 10.1016/j.dib.2023.109446

**Published:** 2023-07-24

**Authors:** Piotr Ciżkowicz, Michał Ledóchowski, Andrzej Rzońca

**Affiliations:** a Warsaw School of Economics, Al. Niepodleglosci 162, 02-554, Warsaw, Poland; b Warsaw School of Economics, Poland

**Keywords:** Fiscal policy, Fiscal consolidations, Fiscal expansions, Narrative studies, Government expenditure, Taxation

## Abstract

The dataset encompasses discretionary fiscal actions in the group of 11 EU New Member States (NMS), which so far have been under-researched, spanning from 2004 to 2019. It extends the narrative dataset of fiscal actions in Advanced Economies created by [1]. Information on actions and their estimated fiscal effects was collected from annually published Convergence or Stability Programmes. Individual actions were classified according to the relevant category of government expenditure and revenue (in line with the classification proposed by [1]) and identified as either exogenous or endogenous (based on the distinction introduced by [3]). The raw data were then aggregated into fiscal plans, by country and announcement year. Finally, two spreadsheets were created. The baseline spreadsheet contains only fiscal consolidation episodes, following the method of [1], while the alternative dataset includes both consolidation and expansion fiscal plans, which extends the methodological approach of [1]. Moreover, in the alternative dataset, the time span of fiscal effects is extended from 5 to 8 years (the longest available) and dividends are added. Dividends represent a category of public revenue not included in [1], but they are non-negligible in the case of the NMS due to the post-socialist legacy.

**Specifications Table**

**Table:** 

Subject	Macroeconomics
Specific subject area	Fiscal policy (narrative identification of fiscal impulses, both consolidations and expansions).
Type of data	Table (Excel spreadsheet).
How the data were acquired	Manual extraction from two main kinds of source documents published annually: Convergence or Stability Programmes of the EU New Member States (additionally, data were acquired from Pre-accession Economic Programmes of Croatia)
Data format	Mixed (raw and aggregated).
Description of data collection	Information on discretionary fiscal measures and their estimated fiscal effects in the EU New Member States was gleaned from annually published Convergence or Stability Programmes. Then, the fiscal effects were rescaled by GDP and aggregated into fiscal plans by category of government expenditure or revenue, country, and announcement year.
Data source location	Convergence or Stability Programmes of following EU Member States published between 2004 and 2019: Poland, Czechia, Slovakia, Lithuania, Latvia, Estonia, Hungary, Romania, Bulgaria, Slovenia, Croatia; Pre-accession Economic Programmes of Croatia published between 2004 and 2013 Programmes can be accessed via WWW: https://commission.europa.eu/business-economy-euro/economic-and-fiscal-policy-coordination/european-semester/european-semester-timeline/national-reform-programmes-and-stability-or-convergence-programmes_en https://ec.europa.eu/economy_finance/economic_governance/sgp/convergence/programmes/index_en.htm https://mfin.gov.hr/highlights-2848/croatia-and-the-eu/economic-programs-within-the-eu/2905
Data accessibility	Repository name: Mendeley data. Data identification number: 10.17632/5xnhy8dxmh.2 Direct URL to data: https://data.mendeley.com/datasets/5xnhy8dxmh/2 Our dataset comprises two spreadsheets: Baseline dataset: DataStructuring consistent with Alesina.xlsx (dataset of fiscal consolidations fully consistent with [1]) Alternative dataset: DataStructuring symmetric.xlsx (dataset of fiscal impulses – both fiscal consolidations and expansions)

## Value of the Data

• The data provide a comprehensive account of discretionary fiscal actions in the EU New Member States. No such dataset is accessible from official sources.
• The dataset is compatible with [1]. Thus, it allows the extension of the study by [1] from advanced economies to the EU New Member States, which so far have been under-researched.
• The data can be used for descriptive analysis and comparisons of fiscal policy across countries and over time. Both fiscal consolidations and expansions can be studied.
• The narrative, action-based dataset can be used in econometric calculations of, for example, fiscal multipliers, which are more accurate than those based on other measures of fiscal impulses.
• In particular, the dataset facilitates analyses of fiscal policy announcements separately from their implementation, thus overcoming the fiscal foresight problem.
• The dataset allows very detailed analyses of the composition of fiscal plans adopted in the EU New Member States.

## 1 Objective

The objective underlying the creation of the dataset is to extend the work of [1] by building a narrative, action-based dataset of fiscal consolidations in the EU New Member States, which have been under-researched. Our dataset adds to the strand of narrative studies of fiscal policy (e.g., [1,^2^,^3^,4]). We seek to produce a database facilitating the analysis of fiscal actions in the EU New Member States, striving for maximum compatibility with the original dataset [1], so the two datasets can be easily merged. Our dataset allows analysis of not only fiscal consolidations but also expansions, unlike [1].

## 2 Data Description

The dataset provided comprises an account of exogenous discretionary fiscal actions in eleven European Union New Member States (Poland, Czechia, Slovakia, Hungary, Lithuania, Latvia, Estonia, Slovenia, Romania, Bulgaria, and Croatia) over the period from 2004 to 2019. Data attached to the article include two spreadsheets: the baseline dataset (DataStructuring consistent with Alesina.xlsx) and the alternative dataset (DataStructuring symmetric.xlsx). The former seeks maximum consistency with the original publication of [1], so only fiscal actions announced during fiscal consolidation episodes are included in the final aggregation. The latter encompasses all exogenous fiscal actions, thus both consolidation and expansion episodes. Both spreadsheets contain worksheets for Macro, Input, and Structured. In the Macro sheet, GDP values and dummies for consolidation years (in the case of the baseline dataset) are included. The Input sheet consists of exogenous fiscal (both deficit-decreasing and deficit-increasing) measures with their announcement year, description, category of taxes/spending, source document, and estimate of fiscal effect (extracted from the source document). In the baseline dataset, only measures pertaining to the tax and spending categories included in [1] are listed, while the alternative dataset additionally includes measures concerning dividends from public property (which, in the case of the EU New Member States, can be non-negligible, given their post-socialist legacy). Consistent with [1], in the baseline dataset, fiscal effects for up to 5 years after the announcement are included, whereas in the alternative dataset, fiscal effects up to 8 years following the announcement are accounted for (the longest horizon available in the source documents). All fiscal effects in the Input sheet are provided in terms of billions of national currency units, on a year-on-year basis (as in [1]). In the Scaled worksheet, fiscal effect estimates are rescaled by the nominal GDP of the year preceding their announcement, consistent with the approach adopted by [1]. In the Structured worksheet, rescaled fiscal effects are aggregated by country, category, and announcement year. The baseline dataset also includes a Final worksheet, which contains aggregated fiscal effects from the Structured worksheet, albeit only for consolidation years.

Both the baseline and alternative datasets are built upon the spreadsheet DataStructuring.xlsx from the original dataset attached to [1]. The data from [1] have been deleted from our datasets, though they can be easily merged, particularly in the case of our baseline dataset.

A detailed description of the Excel spreadsheets with our dataset is provided in Table 1 below. Table 2 contains a description of how measures are classified into categories of public spending and revenue.

**Table 1:** Detailed information on the dataset.

Worksheet	Variable name	Variable description	Notes	Spreadsheet
Macro	Country	Country name	Country code in the adjacent column	Baseline and alternative
	Year	Year, to which GDP and consolidation_dummy are referred		Baseline and alternative
	GDP	Nominal GDP in national currency billions for a given year (in the cases of NMS which joined the Euro Area prior to 2019, GDP in euros from the year before the euro adoption onwards, and in national (pre-euro) currency beforehand)		Baseline and alternative
	consolidation_dummy	1 if the fiscal consolidation episodes occurs in the given year, 0 otherwise	Fiscal consolidation occurs in a given year, if and only if, the sum of the exogenous fiscal consolidation measures announced in this year or in the preceding years is greater than the sum of exogenous and endogenous fiscal expansion measures announced in this year or in the preceding years, like in [1]	Baseline only

Input	Country	Two-letter country code		Baseline and alternative
	Year	Announcement year of fiscal measure		Baseline and alternative
	Category	Category of fiscal expenditure/revenue which the measure concerns (code)	Consistent with classification used by [1], in alternative dataset category of Dividends added, see Table 2	Baseline and alternative
	Components	Category of fiscal expenditure/revenue which the measure concerns	Consistent with classification used by [1], in alternative dataset category of Dividends added, see Table 2	Baseline and alternative
	Measure	Description of the measure		Baseline and alternative
	Impact *t* + *x*	Fiscal effect of the measure in a year x following its announcement, in national currency billions (for countries which adopted euro prior to 2019 in euros from the year of year adoption onwards). Fiscal effect is the change in general government balance on a year-on-year basis arising from the respective legislative measure (positive if measure improves fiscal balance, negative otherwise). All fiscal effect estimates are extracted from the source documents.	In baseline dataset fiscal effects are provided up to 5 years after the announcement as in [1] dataset. In alternative dataset fiscal effects are provided up to 8 years after the announcement (the longest available time span)	Baseline and alternative
	Source	Source document whence information on fiscal measure and estimated fiscal effect were extracted		Baseline and alternative
Scaled	Label	Concatenation of columns Country, Year and Category from Input sheet		Baseline and alternative
	scaled (*t* + *x*)	Fiscal effect of the measure in a year x following its announcement (from Input sheet), % of GDP of the year preceding its announcement	In baseline dataset fiscal effects are provided up to 5 years after the announcement as in [1] dataset. In alternative dataset fiscal effects are provided up to 8 years after the announcement (the longest available time span)	Baseline and alternative
	unscaled (*t* + *x*)	Fiscal effect of the measure in a year x following its announcement (from Input sheet), billions of national currency	In baseline dataset fiscal effects are provided up to 5 years after the announcement as in [1] dataset. In alternative dataset fiscal effects are provided up to 8 years after the announcement (the longest available time span)	Baseline and alternative

Structured	Country	Two-letter country code		Baseline and alternative
	Year	(Announcement) year to which aggregated fiscal affects are referred		Baseline and alternative
	u_t	Aggregated instantaneous fiscal effect of measures announced in a given year (in terms of % of GDP of year preceding the announcement of respective measure)	For various categories of spending and taxes and total spending and taxes (row 1)	Baseline and alternative
	a_t	Aggregated fiscal effect for a year t(column Year) of measures announced in preceding years (in terms of % of GDP of year preceding the announcement of respective measure)	For various categories of spending and taxes and total spending and taxes (row 1)	Baseline and alternative
	a_*t* + *x, x*>0	Aggregated fiscal effect for a year t(column Year)+*x* of measures announced in the year t and preceding years (in terms of % of GDP of year preceding the announcement of respective measure)	For various categories of spending and taxes and total spending and taxes(row 1). In DataStructuring consistent with Alesina.xlsx fiscal effects are provided up to 5 years after the announcement as in [1] dataset. In DataStructuring symmetric.xlsx fiscal effects are provided up to 8 years after the annoucement (the longest available time span)	Baseline and alternative
	Tax-Impact Tot	Aggregated fiscal effect for year t of tax measures announced in a given year and preceding years (in terms of % of GDP of year preceding the announcement of respective measure)		Baseline and alternative
	Spending Impact Tot	Aggregated fiscal effect for year t of expenditure measures announced in a given year and preceding years (in terms of % of GDP of year preceding the announcement of respective measure)		Baseline and alternative
	Direct Tax/Indirect Tax/NYC Tax/Other Tax/Cons-Inv/Transfers/NYC Spend/Other Spend/Tax/Spending Tot	Aggregated fiscal effect for year t and subsequent years of measures announced in a given year and preceding years, for subsets of measures pertaining to various categories of spending and taxes and total spending and taxes (respectively)	Direct taxes comprise income and property taxes, Cons-Inv encompasses public consumption, investment and public sector salaries spending	Baseline and alternative

Final	Country	Two-letter country code		Baseline only
	Year	(Announcement) year to which aggregated fiscal affects are referred		Baseline only
	consolidation_dummy	consolidation_dummy variable from Macro sheet		Baseline only
	Other variables	Respective variables from Structured sheet multiplied by consolidation_dummy		Baseline only

Source: [1], own elaboration.

**Table 2:** Classification of fiscal measures.

Category	Components	Description	Tax/Spending	Note
CONS	Consumption	Consumption	Spending	
INV	Investment	Investment	Spending	
SAL	Salaries	Salaries	Spending	
UNEM	Transfers	Unemployment	Spending	
HLT	Transfers	Health Related	Spending	
EDU	Transfers	Education	Spending	
PENS	Transfers	Pensions	Spending	
FIRSUB	Transfers	Firm Subsidies	Spending	
FCPO	Transfers	Family and Children Policies	Spending	
RD	Transfers	Research&Development and Firm	Spending	
OTSUB	Transfers	Other Subsidies	Spending	
OTHSS	Transfers	Other Social Security	Spending	
NYCEX	NYC Spending	Not Yet Classified Spending	Spending	
OTHEX	Other Spending	Other Spending	Spending	
PIDT	Personal Income Tax	Personal Income Direct Taxes	Tax	Including social contributions paid by employee
TCDPT	Personal Income Tax	Tax Credits and Deductions - Private	Tax	
CDT	Corporate Tax	Corporate Direct Taxes	Tax	Including social contributions paid by employer
TCDCP	Corporate Tax	Tax Credits and Deductions - Corporate	Tax	
NYCPC	NYC CvsP	Not Yet Classified Corporate vs Private (Direct Taxes)	Tax	
TCDNYC	NYC CvsP	Tax Credits and Deductions - NYC Corporate vs Private	Tax	
PROPPT	Property Tax Private	Property Taxes - Private	Tax	
PROPCP	Property Tax Corporate	Property Taxes - Corporate	Tax	
PROPNYC	Property Tax NYC	Property Taxes - NYC Corporate vs Private	Tax	
INDT	Goods and Services	Indirect Taxes	Tax	
TCDIND	Goods and Services	Tax Credits and Deductions - Indirect	Tax	
OTHTX	Other Tax	Other Taxes	Tax	
NYCTX	NYC Tax	Not Yet Classified Taxes	Tax	
DIV	Dividends	Dividends	Tax	Dividends and other public property income, only in alternative dataset

Source: [1], own elaboration.

## 3 Experimental Design, Materials and Methods

The data collection methodology largely builds upon the earlier work by [1]. Information on fiscal measures and their estimated fiscal effects was extracted from annual Convergence or Stability Programmes (Plans) submitted by the governments of the respective EU member states. For the purpose of data collection, Plans issued between 2004 and 2019 were considered (in the cases of Romania and Bulgaria, plans were published only since these countries' EU accession in 2007; in the case of Croatia, Pre-accession Economic Programmes issued between 2004 and 2012 and the Economic Programme of 2013 were also taken into account). Measures announced prior to 2004 were only taken into account if included in later Programmes. Announcement years of the individual measures were also determined as provided in the Plans; if no precise information was provided, it was generally assumed that the announcement year was consistent with the year when the respective Programme was published.

Fiscal effect estimates were gleaned from the Plans, and if provided in terms of GDP percentage, converted into national currency units based upon GDP figures and forecasts available in the respective Plans. If estimates of fiscal effects were provided in the Plan in a currency different from the national currency of a given country in the year the Plan was published, they were converted using the annual average exchange rate in the given year (such cases were very rare). Sometimes, information on fiscal measures with no attached estimates of fiscal effects was provided in the Plans, and these measures had to be disregarded for practical purposes, since our dataset is of a quantitative nature. If the Plan stipulated that a measure had come into force during the course of the year and only a full-year fiscal effect was provided, we assumed that the fiscal effect was distributed proportionally across the year.

Classification of fiscal measures into tax and spending and various subcategories was done based on the information provided in the Plans, following the approach of [1]. When the available information was not sufficient to definitively categorize the measure into any of the existing categories, they were classified as Not Yet Classified (Taxes or Spending). Additionally, the measures were classified as either endogenous or exogenous, following the distinction proposed by [3], and applied by [1]. Endogenous measures, in line with [3], are aimed at counteracting shocks affecting output. They include primarily countercyclical fiscal stimulus or retrenchment and tax increases enacted to finance new expenditure. Exogenous fiscal measures are those that are not linked to the current state of the economy and aim at, for example, boosting long-term economic growth or public debt correction [1], [3]. The distinction between endogenous and exogenous measures is based on the underlying motives, as provided in the Plans. We pursued a conservative approach, thus measures are classified as exogenous, unless available evidence suggests otherwise. All announced measures were treated as fully credible; thus, any subsequent amendments or withdrawals of measures entered our dataset as new fiscal actions.

The collected data was then aggregated into fiscal plans, following the approach of [1] and using the spreadsheet DataStructuring.xlsx attached to their book. When constructing our baseline aggregated dataset, we determined fiscal consolidation years as years when the sum of exogenous deficit-decreasing measures is greater than the sum of both exogenous and endogenous deficit-increasing measures (all measures announced in a given year or in the preceding years, if their fiscal effects have not yet materialized, are taken into account). Only exogenous fiscal measures during consolidation episodes are finally aggregated, in line with [1]'s approach. Moreover, in the aggregation process, the fiscal effects for horizons longer than 5 years from the announcement year are disregarded, even if estimates are provided in the source documents, and measures concerning dividends from public property are dropped since they do not appear in the original [1] dataset.

In constructing the alternative, symmetric dataset (DataStructuring symmetric.xlsx), a few departures from [1] are made. First, no consolidation episodes are identified, and all (both deficit-increasing and deficit-decreasing) exogenous fiscal measures are used for aggregation. Second, fiscal effects up to 8 years after the announcement (the longest horizon available in the Plans) are taken into account. Finally, an additional category of public revenue (Dividends) covering measures pertaining to dividends and other income from public property is added. Thus, the symmetric dataset, while not fully consistent with [1], makes greater use of available data. Just like in the case of the baseline dataset, the spreadsheet DataStructuring.xlsx attached to the book [1] is used for the purpose of aggregation.

## Ethics Statements

The authors declare that they comply with all the ethical guidelines as stated in Data in Brief's Guide for Authors. Authors declare that they did not conduct human or animal studies and did not need permission to use primary data.

## CRediT authorship contribution statement

**Piotr Ciżkowicz:** Conceptualization, Supervision, Validation, Writing – review & editing. **Michał Ledóchowski:** Investigation, Formal analysis, Writing – original draft. **Andrzej Rzońca:** Conceptualization, Supervision, Validation, Writing – review & editing.

## Data Availability

Narrative dataset of fiscal actions in the EU New Member States (Original data) (Mendeley Data).

## References

[1] Alesina A., Favero C., Giavazzi F. (2019).

[2] P. Devries, J. Guajardo, D. Leigh, A. Pescatori, *A new action-based dataset of fiscal consolidation*, IMF Working Paper No. 11/128, (2011).

[3] Romer C., Romer D. (2010). The macroeconomic effects of tax changes: estimates based on a new measure of fiscal shocks. Am. Econ. Rev..

[4] Vegh C., Vuletin G. (2015). How is tax policy conducted over the business cycle?. Am. Econ. J..

